# Physical healing as a function of perceived time

**DOI:** 10.1038/s41598-023-50009-3

**Published:** 2023-12-17

**Authors:** Peter Aungle, Ellen Langer

**Affiliations:** Psychology Department, Harvard University, Cambridge, USA

**Keywords:** Human behaviour, Psychology, Signs and symptoms

## Abstract

In this study we wounded study participants following a standardized procedure and manipulated perceived time to test whether perceived time affected the rate of healing. We measured the amount of healing that occurred across three conditions using a within-subjects design: Slow Time (half as fast as clock time), Normal Time (clock time), and Fast Time (twice as fast as clock time). Based on the theory of mind–body unity—which posits simultaneous and bidirectional influences of mind on body and body on mind—we hypothesized that wounds would heal faster or slower when perceived time was manipulated to be experienced as longer or shorter respectively. Although the actual elapsed time was 28 min in all three conditions, significantly more healing was observed in the Normal Time condition compared to the Slow Time condition, in the Fast Time condition compared to the Normal Time condition, and in the Fast Time condition compared to the Slow Time condition. These results support the hypothesis that the effect of time on physical healing is directly affected by one’s psychological experience of time, independent of the actual elapsed time.

## Summary paragraph

The theory of mind-body unity posits bidirectional and simultaneous influences of mind on body and body on mind^1^. In some domains, these dynamics are increasingly well understood. Research into psychological influences on chronic pain, emotion and physiological health, and placebo effects—particularly those involving administration of inert medications—have led to meaningful improvements in treatments for a broad range of illnesses and injuries. Mindfulness based stress reduction and cognitive behavioral therapies are now considered first-line treatments for chronic pain^2^; clinicians increasingly seek to address the role of chronic stress when treating everything from high blood pressure to cardiovascular risks^3^; and factors shown to amplify or attenuate placebo effects are gradually being applied to improve conventional treatment outcomes^4^. However, the breadth of psychological influences on physical health remains understudied. Here we show that the effect of time on physical healing is significantly influenced by the psychological experience of time. Compared to a control condition, in which perceived time and actual time were equal, we found that experimentally induced wounds healed faster when participants believed more time had passed and slower when they believed less time had passed, even though the actual elapsed time was always the same. Our results demonstrate that the effect of time on physical healing is inseparable from the psychological experience of time. By placing these results within the context of other surprising mind-body unity phenomena (e.g.,^5–8^), we hope to encourage other researchers in the field to expand the scope of factors considered when investigating psychological influences on health and well-being.

## Introduction

Open Google Scholar and start typing “mind body” into the search box. The top two suggested search terms are “mind body problem” and “mind body connection.” Select “mind body connection,” and Google Scholar returns more than 5 million results. The top results link to papers that cover the topics of emotion^9^, chronic pain^10^, and placebos^11^. In some domains, the simultaneous and bidirectional influences of mind on body and body on mind are intuitive and increasingly well-studied, as indicated by the 5 million results returned on Google Scholar. But in other domains, the ability of the mind to shape physical health is overlooked, discounted, or rejected altogether. “Mind–body connection” is the most commonly used phrase to describe mind–body effects on health, but using the word “connection” to broadly describe this line of research suggests a narrower range of domains to which they apply than do words like “system” or “unity,” which emphasize the artificiality of brain–body distinctions.

For example, genetic predispositions are generally considered to be unamenable to psychological intervention–behavioral interventions perhaps, but not purely psychological interventions. Although genotypes are distinguished from genotypic expression (phenotypes), the factors affecting phenotypic expression—e.g., diet, temperature, oxygen levels, humidity, light cycles, the presence of mutagens—generally do not consider mental processes. A study by Turnwald et al.^12^, however, suggests mindsets matter. In one experiment, they provided half of the participants with false feedback about whether the participants had genotypes that predisposed them to high or low exercise capacity, defined physiologically in terms of CO_2_O_2_ exchange rate, maximum ventilatory physiology, and total run time. In a second experiment, they provided half of the participants with false feedback about whether they had genotypes that predisposed them to high or low thresholds for satiety, defined physiologically in terms of changes in glucagon-like peptide 1 (GLP-1) after eating. In both experiments, the observed physiological changes corresponded to the genotypic feedback participants received, regardless of whether it was true or false: physiological correlates of exercise capacity and satiety were predicted by the feedback participants received, not their actual genotypes^12^. Similarly, the benefits of exercise almost by definition would seem to require the act of *exercising*, or at the very least an increase in physical activity or change in diet. If a person who does not exercise weighed themselves, checked their blood pressure, took careful body measurements, wrote everything down, maintained their same diet and level of physical activity, and then repeated the same measures a month later, few would expect exercise-like improvements. But in a study involving hotel housekeepers, that is effectively what the researchers found^13^. Half of the housekeepers were informed their work meets the federally recommended guidelines for daily physical exercise and half were not. The group that received that information lost weight, had lower blood pressure, and slimmer waist-to-hip ratios compared to the group that did not, despite no change in workload, exercise habits, overall physical activity, or diet in either group. These results are consistent with more recent research demonstrating that Activity Adequacy Mindsets (AAMs)—whether people believe their level of physical activity is sufficiently healthy—affect physiological measures such as resting heart rates and blood pressure independent of actual physical activity^14^.

Aging is another example. Scientists typically define aging as, “time-related deterioration of the physiological functions necessary for survival and fertility”^15^—i.e., deterioration that is a function of an external constant—time—that is independent of related underlying mental representations and processes. This view of aging, however, risks mindsets that may become self-fulfilling. For instance, in a longitudinal study designed to test whether negative aging stereotypes formed when study participants were younger were associated with higher odds of experiencing cardiovascular events when they were older, researchers found that negative aging stereotypes—even after controlling for age, body mass index, cholesterol, depression, education, elevated blood pressure, family history of cardiovascular death, gender, marital status, number of chronic conditions, race, self-rated health, and smoking history—predicted significantly higher odds of experiencing cardiovascular events as participants aged^16^. The effects of mindsets on physical health and wellbeing can operate in the reverse direction too. In the first study of mind–body unity, we recruited elderly men between the ages of 70 and 75 for “a study about reminiscing,” assessed each participant on a range of physical health and cognitive competency measures, then took them on a retreat to a remote monastery in New Hampshire that we had retrofitted with furniture, appliances, periodicals, and so on from 20 years earlier. The men were divided into two groups that followed nearly identical procedures—namely to use the retreat as an opportunity to mentally inhabit the past, which we suggested could make them feel as well as they had then. The instructions for one of the groups went further, however, by having participants not only reminisce about the past but to live as if it were 1959 (i.e., 20 years earlier), meaning no discussion of anything that happened after September 1959 and “letting [themselves] be just who [they] were in 1959.” Relative to baseline measures, participants in both groups experienced significant improvements in measures of physical health and cognitive performance: hearing, vision, memory, joint flexibility, and hand strength improved, and judges blind to the study design and hypothesis believed participants looked significantly younger in photos taken after the retreat compared to photos taken before. Participants in the group instructed to live as their 1959 selves saw even greater improvements in joint flexibility, as well as improvements in manual dexterity and cognitive performance^17^. Both studies illustrate the bidirectional effects of mental states on a physiological process, specifically aging, not ordinarily believed to be influenced by how the process is mentally construed.

Studying mind–body dynamics to better understand chronic pain, emotion physiology, and placebos is important, but thinking in terms of phenomena obviously related to psychological factors perhaps explains why rigorous research on the broader influences of mental states on physical health remains comparatively scarce. Even results from the most novel, surprising, and unconventional placebo research (e.g.,^18–24^) often still seem to suggest implications that are limited to psychological influences on medical treatments and illness trajectories among patients who are already sick. But mind–body unity suggests that perceptions, expectations, beliefs, and so on—acquired through interaction with the world—are continuously affecting vision, aging, healing, and other biological and physiological processes that determine whether one develops an illness or requires medical treatment in the first place. Expectations influence more than the physiological responses to emotions, pain, and placebos. For example, simply expecting fatigue can actually cause one to become fatigued^25^; simply expecting to be able to see can actually improve one’s vision^26^; simply expecting to catch a cold is associated with significantly higher odds of actually catching one^27^.

Few studies have explored whether perceived time affects physiological processes independently of actual time. If the physiological effects of time are unaffected by the subjective experience of time, all else equal, perceived time should not materially affect physiological outcomes. Mind–body unity suggests otherwise: human time perception requires concepts of time. As we acquire concepts through learning and experience, they become embedded within networks of related expectations, physiological reactions, and beliefs (e.g.,^28–30^). Manipulating perceived time should activate the same brain-body networks as the actual passage of time. Results from the few studies on perceived time and physiological function that do exist support this idea. For example, blood glucose levels have been shown to follow perceived time, not actual time^31^, as have reaction times and EEG activity following perceived sleep deficits, not actual sleep deficits^32^. The purpose of this study was to test whether manipulating perceived time, while holding real time constant, would affect physical healing. More specifically, we hypothesized that experimentally induced wounds would heal faster when more perceived time had passed and heal slower when less perceived time had passed, despite no differences in actual elapsed time.

## Experimental design overview

We wanted a salient way to measure healing, and previous research has found the skin is quite responsive to expectations. For example, patients who received physician assurances after skin pricks healed significantly faster^33^, and the suggestion that one had touched poison ivy resulted in stronger symptoms than actually touching poison ivy^34^. This experiment tested whether cupping marks produced by identical cupping treatments healed faster or slower as a function of perceived time. That is, would the cupping marks heal slower when perceived time was slower and faster when perceived time was faster, despite equivalent elapsed time across conditions?

To test our hypothesis, we recruited participants for a study on “the relationship between personality and healing after cupping therapy.” Cupping is a technique that has been used for centuries to help with muscle tension and pain relief^35^. Cupping therapy involves creating a localized suction on the skin using cups, which causes the expansion of blood vessels beneath the skin. This process leads to increased blood flow and the accumulation of blood and fluids in the area, resulting in bruising post-therapy (see Supplementary Materials). It was used in this study, not for any therapeutic purpose, but rather because of the incidental marks left on the skin after cupping treatments, which allowed for a standardized process to create wounds that healed within an appropriate timeframe to observe the process experimentally. Participants completed several personality and psychosocial measures of wellbeing at their time of enrollment to maintain our cover story while also allowing subsequent analyses to account for factors such as stress, anxiety, and depression known to be related to wound healing^36^. A three-item measure designed to assess participants’ subjective sense of how quickly they tend to recover after an injury was also included. Our hypothesis was that perceived time would significantly predict healing even though elapsed time was held constant.

Research has demonstrated that participants sense of time over relatively short periods can be significantly swayed by the temporal feedback they receive^37^. To account for individual variability in responsiveness to the cupping procedure, a within-subjects design was used. Across three conditions participants spent the same amount of real time (28 min) monitoring changes in cupping mark wounds produced by a standardized cupping procedure; perceived time in two of the conditions was altered using a manipulated timer such that perceived time was equal to half (14 min) or twice (56 min) the amount of real time. In all three conditions, participants completed another task after the healing observation period in order to balance perceived time with actual time and ensure each lab session lasted for approximately one hour, as advertised. Because participants could have based their assessments of healing on perceived time, given the assumption that healing is often a function of time, we obtained independent judgments of healing from raters blind to our hypothesis and study conditions. We hypothesized that healing would be greatest in the Fast Time condition and least in the Slow Time condition.

## Materials and methods

Thirty-three participants (average age = 28.33 years old, SD = 7.78 years; 23 women; 55% White, 18% Asian, 9% Black or African American, 9% Hispanic or Latino, 9% mixed ethnicity) were recruited from The Harvard Public Study Pool for a study on personality and healing after cupping therapy. Participation lasted between 10 and 14 days, depending on how quickly each participant completed the three in-lab sessions, each of which had to be completed on separate days. The first in-lab session could only be completed after the participant finished a weeklong series of at-home exercises (described in more detail below).

All participants completed the three experimental conditions: Slow Time (14 min), Normal Time (28 min), and Fast Time (56 min). The Normal Time condition was based on standard clock time and served as the control condition. Condition order was counterbalanced across participants prior to beginning the study. The location of the cupping procedure during the lab sessions varied by lab session: Session 1 applied the cupping procedure to the upper part of participants’ nondominant forearm, Session 2 applied it to the midpart of the forearm, and Session 3 applied it to the lower part of the forearm. By varying the location of the cupping treatment in the same way for all participants while counterbalancing the condition order, the procedure separated the effect of condition from the effect of slight variations in the location of the treatment. All experimental procedures were reviewed and approved by the Harvard Institutional Review Board’s Committee on the Use of Human Subjects (IRB22-0237), and all methods were performed in accordance with the relevant guidelines and regulations.

Participants were paid $10 for each component of the study they completed, plus $5 Amazon Gift Cards after completing the full study. Participants who completed the study also received entry into a raffle offering five prizes of $50 Amazon Gift Cards, to be awarded once data collection was completed. In order to participate, participants had to be between 18 and 50 years of age; free of tattoos, rashes, or other marks on the bottom side of their non-dominant forearms; not currently taking any anti-coagulant or blood-thinning medications; and never diagnosed with severe anemia, hemophilia, or diabetes. Following screening, we obtained informed consent, had participants complete an enrollment survey, and provided them with cupping kits for the at-home exercises. The enrollment survey collected demographic information and assessed anxiety and depression using the hospital anxiety and depression scale^38^, the big five personality traits using the ten-item personality inventory^39^, chronic experiences of stress using the perceived stress scale^40^, and mindfulness using the Langer mindfulness scale^41^.

### At-home exercises

Participants repeated the following exercise once each day for one week prior to their first in-lab session: “Place the cup in the middle of the bottom side of your non-dominant forearm and attach the vacuum pump. Squeeze the pump 5 times. After 5 pumps, wait for 30 s. Remove the cup, take a photo, then click on the first Qualtrics link and complete the survey. After 30 min, take another photo, click to the next page of the survey, complete the same items, and upload the second photo.” This was identical to the cupping procedure that was applied by the experimenter during their three lab sessions. As participants looked at the mark and completed both surveys, we asked them to notice the discoloration, intensity, and severity of the cupping mark. The purpose of these exercises was to create conditioned expectations of healing within a 30-min timeframe, approximately equal to the 28-min healing observation period they would experience during their three lab sessions.

### Normal time condition

Perceived time and actual time of the healing observation period were equal to 28 min in this condition. When participants came into the lab, we collected their personal belongings, including phones, watches, and any other electronic devices they had with them, which we told them was “to make sure they can avoid distractions.” Their belongings and devices were returned to them at the end of each lab session. The experimenter then seated the participant in front of a desktop computer where they could complete the healing surveys. A digital timer was displayed on a tablet next to the desktop computer (see Supplementary Materials). The timer displayed elapsed time in minutes only. Participants were told, “We are varying a number of things between lab sessions to ensure we have a robust measure of healing, including how long we observe the process, the intervals between observations, and the location of the cupping treatment. The cupping procedure itself and the number of times you complete the healing survey (7 times) will remain the same during all three lab sessions.” Following this statement, the experimenter explained that the participant would play Tetris in between healing surveys, and that the participant would need to monitor the timer on the tablet to make sure they completed each healing survey at the right interval, which, in the Normal Time condition, was once every 4 min. The timer screen turned green for 1 min at each survey interval to indicate it was time to complete the next survey. The timer ran continuously to prevent the addition of extra time to the healing observation period that would result from resetting the timer after each survey. After confirming the participant understood the instructions, the experimenter placed a 1.5″ diameter cup on either the upper (Session 1), middle (Session 2), or lower portion of the participant’s non-dominant forearm (Session 3), attached the vacuum pump, squeezed the pump 5 times, set a timer for 30 s, and removed the cup when the timer went off. The experimenter then had the participant place their arm within a rectangle marked by masking tape, forearm turned up, and took a photo using an iPhone 12 Pro Max attached to a ring light. A light meter was used to ensure a standardized light intensity of approximately 250 Lux for all photos^42^. Every 4 min, participants completed the healing survey measures, which asked them to rate how intense, irritated, severe, discolored, and visible the cupping mark appeared on 10.0 scales. They were also asked to rate the amount of healing that had taken place on a 10.0 scale, and how happy, restless, and stressed they felt at that moment on a 6-point Likert scale. Having participants monitor the timer in order to complete the healing surveys ensured that they attended to the passage of time. At 28 min, the experimenter took another photo following the same procedure described above, then asked participants if they would feel comfortable following the same procedure on the bottom of their *dominant* forearm after they finished the 7^th^ survey. It was emphasized that this portion of the experiment was optional. This latter portion of the procedure was included to maintain the cover story that each session would last approximately one hour.

### Fast time condition

Perceived time for the healing observation period was 56 min, while clock time was 28 min. The experimenter followed a procedure identical to that described above for the Normal Time condition, except participants were instructed that for this session, they would complete the healing survey every “8 min.” Perceived time was manipulated by altering the tablet timer to run at twice its normal speed. After the 7^th^ survey, participants were told we were piloting several TV clips for another study and would like them to rate each clip to help us select the best one. The clips they rated were selected because they were rated among the most engaging and absorbing on YouTube, and highly engaging activities decrease subjective duration^43^. The purpose of decreasing subjective duration in this step was to counteract the Fast Time manipulation so that by the time participants were dismissed for the day, perceived time and clock time balanced out to just over one hour.

### Slow time condition

Perceived time for the healing observation period was 14 min, while clock time was 28 min. The experimenter followed a procedure identical to that described above for the Normal Time condition, except participants were instructed that for this session, they would complete the healing survey every “2 min.” Perceived time was manipulated by altering the tablet timer to run at half its normal speed. After the 7^th^ survey, participants played “a game” for 15 min that created a high cognitive load (based on the task used in^44^), which has been shown to increase retrospective subjective duration judgments^45^. The purpose of increasing subjective duration in this step was to counteract the Slow Time manipulation so that by the time participants were dismissed for the day, perceived time and clock time balanced out to approximately one hour.

## Results

Psychology study participants—college students in particular—are notorious for trying to guess the true hypotheses of the studies they participate in^46,47^, so at the end of the third lab session, we asked participants to guess what they thought the study was ultimately about. We wanted to see if the manipulated timer was salient enough to influence how participants answered this question, regardless of whether they correctly guessed the study hypothesis. About half of the participants thought we were interested in how mood, stress, or attention related to healing, and about half thought that we were interested in factors that influence *subjective* perceptions of healing. While it would have been interesting to observe the predicted effect even if participants correctly guessed our hypothesis, their responses indicated they did not.

Two participants did not complete all three lab sessions. Four participants missed some of the seven healing observation surveys in one of the conditions (1 participant in the 14-min condition and 3 participants in the 56-min condition). Excluding these data did not change the pattern of results reported below (we include models with these exclusions in our Supplementary Materials).

To impartially assess healing, we used a “wisdom of crowds” approach, since it has been shown to result in more accurate estimates than those provided by individual experts (e.g.,^48–50^). We recruited 25 master workers (average age = 49.44 years old, SD = 12.98; 10 women, 14 men, 1 nonbinary) on Amazon’s Mechanical Turk, which allowed for inexpensive and rapid data collection from a subset of users who have a history of providing high quality responses. The exact requirements to be awarded “master worker” status are not disclosed by Amazon, but the designation is given to workers who have high task approval rates, have completed a large and diverse number of tasks, have been active on the platform for a significant period of time, have demonstrated consistent compliance with task guidelines, and have low task rejection rates^51^. They rated the amount of healing that had occurred by comparing the second photo taken during each lab session to the first photo taken. After a brief set of instructions, the workers were presented with randomized pairs of photos and asked to rate healing on a 10.0 scale (0.0 = not at all healed, 5.0 = somewhat healed, 10.0 = completely healed). The figure below displays the average healing rating for each participant plotted by condition. Healing in the 14-min condition had a mean rating of 6.17 (SD = 2.59, 32 Subjects, 800 ratings); healing in the 28-min condition had a mean rating of 6.43 (SD = 2.54, 33 Subjects, 825 ratings); and healing in the 56-min condition had a mean rating of 7.30 (SD = 2.25, 32 Subjects, 800 ratings).

**Figure 1:**
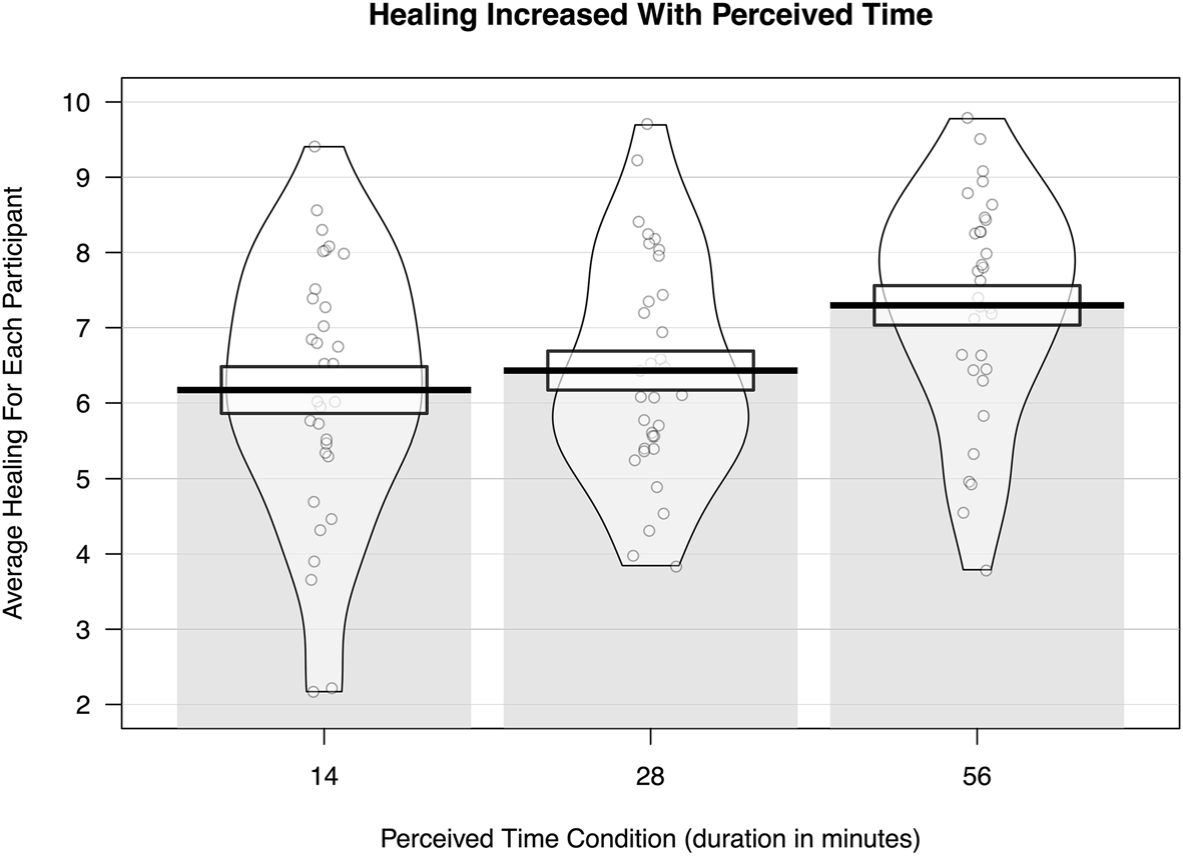
Violin plots of mean healing for each participant within each condition. The more perceived time that passed, the more participants healed on average.

One way to think about the differences in average healing by condition is to think about the number of participants that were judged to have almost completely healed by the end of the session. Only 5 out of 32 participants had mean healing ratings of 8 or above in the 14-min condition, compared to 8 out of 33 participants in the 28-min condition, and 11 out of 32 participants in the 56-min condition. In other words, just over a third of participants had almost completely healed in the 56-min condition—more than double the percentage of participants who had almost completely healed in the 14-min condition (Fig. 1).

A mixed between-within-subjects linear model was constructed to test whether the observed effect of our time manipulation on healing could be attributed to chance. We started with the simplest model in which healing was the outcome variable, study condition was the predictor, and individual rater and subject variability were accounted for by including random intercepts in the model for both subjects and raters. We then added demographic (age, gender, ethnicity), psychosocial (stress, anxiety, depression, mindfulness), contextual (session number, session mood), and personality trait (openness, conscientiousness, extraversion, agreeableness, neuroticism) covariates using a blockwise model selection process. Table 1 presents the output from the model that fit best. Including personality trait variables did not significantly improve the model, and we did not have any theoretical reason to retain them in our analysis. We measured personality traits only to maintain our cover story (we include model outputs for each block in our Supplementary Materials, however).

**Table 1:** Mixed effects outputs for best-fit model.

	Healing estimate	*p*-value
Fixed effects		
“14”-minute condition (intercept)	7.52	** < 0.001**
“28”-minute condition	0.25	**0.013**
“56”-minute condition	1.03	** < 0.001**
Age	− 0.05	**0.009**
Gender (male)	− 0.55	0.099
Ethnicity (white)	− 0.49	0.114
Anxiety	− 0.04	0.44
Stress	− 0.09	**0.01**
Depression	0.14	0.083
Mindfulness	0.02	0.27
Session 2	− 0.03	0.762
Session 3	0.07	0.459
Session mood	0.13	**0.043**
Random effects		
Residual variance	3.50 [SD = 1.87]	
Random intercept variance (subjects)	0.59 [SD = 0.77]	
Random intercept variance (raters)	1.47 [SD = 1.21]	
Intraclass correlation	0.37	
N	33 _Subjects_	
	25 _Raters_	
Observations (conditions × subjects × raters)	2425	
Marginal R^2^/conditional R^2^	0.122/0.447	

Significant values are in bold.

Pairwise contrasts, with p-values adjusted using the Tukey method and degrees of freedom adjusted using the Kenward-Roger method, showed a significant difference in mean healing in the 56-min condition compared to the 28-min condition (Mean difference = 0.779, t(2374) = 7.225, p < 0.0001), a significant difference in the 56-min condition compared to the 14-min condition (Mean difference = 1.03, t(2378) = 10.713, p < 0.0001), and a significant difference in the 28-min condition compared to the 14-min condition (Mean difference = 0.251, t(2374) = 2.487, p = 0.0346).

Ethnicity was condensed into two levels (white, not white). Anxiety and depression estimates are based on scaled scores using the Hospital Anxiety and Depression Scale. Stress estimate is based on scaled scores using the 14-item Perceived Stress Scale. Mindfulness estimate is based on scaled scores using the 14-item Langer Mindfulness Scale. Session mood estimate is based on scaled scores measured during each lab session.

Table 1 includes the parameter estimates and p-values for the fixed effect variables; random intercept variance within each grouping factor (subjects and raters); and residual variance not accounted for by the fixed or random effects we included. The intraclass correlation value of 0.37 reflects the extent to which assessed healing differed between individual subjects across the three conditions. A lower ICC value suggests that there was less variability in healing outcomes between subjects, indicating that the condition effect was relatively consistent across subjects.

## Discussion

The results reported above demonstrate a clear relationship between perceived time and the rate of physical healing. In conditions where time was perceived to pass faster, wound healing was accelerated, whereas slower perceived time led to slower healing. This is an important finding, as it challenges conventional medical wisdom about the influence of psychological factors on physiological outcomes, which typically argues psychological influences only affect health indirectly, primarily by influencing behavior^52,53^. These data are consistent with the theory of mind–body unity, however, which proposes that the mind and body interact in a bidirectional and simultaneous manner. Our results contribute to a growing body of evidence suggesting that abstract psychological precepts, such as those that guide how we perceive the passage of time, can significantly impact physical health outcomes.

Earlier we mentioned that few studies have investigated the effects of perceived time on physiological outcomes, but two studies from our lab have found effects consistent with our results.

In one of those studies, we found that blood glucose levels (BGLs) among diabetics followed perceived time, not clock time^31^. We recruited 47 participants with type 2 diabetes mellitus and randomly assigned them to one of three experimental conditions (Fast, Normal, Slow). Participants in all three conditions played video games for 90 min of real time. They were instructed to switch video games every 15 min to make sure they monitored the passage of time. Prior to coming into the lab, they were instructed to record their BGLs before and after every meal for at least one week, thus establishing a temporally conditioned expectation for fluctuations in their BGLs. Participants in the Fast condition monitored a timer running at 2 × real time, while those in the Slow condition monitored a timer running at 0.5 × real time. The results showed that BGLs changed as a function of perceived time rather than actual time. Those who believed more time had passed (Fast condition) showed a greater decrease in BGL compared to those in the Normal group. Conversely, those who perceived less time had passed (Slow condition) showed a lesser decrease in BGL.

In the other study, we found that EEG activity and cognitive performance on a memory recall test followed perceived sleep rather than actual sleep^32^. Sixteen healthy participants (8 females, average age 24.2 years) were given 8 h of sleep on the first night and 5 h on the second night. Upon waking, they were randomized to believe they had either 8 or 5 h of sleep using a manipulated clock. Prior to beginning the study, they were required to maintain a sleep/wake diary for 3 weeks, as well as an actigraphy for at least one week, arguably also creating a conditioned expectation relevant to the study design and hypothesis. Cognitive performance was evaluated using auditory psychomotor vigilance tests, subjective sleepiness ratings, and waking electroencephalography (EEG). The findings revealed that reaction times were significantly quicker when participants thought they had 8 h of sleep after only 5 h, and conversely, slower when they believed they had 5 h of sleep after actually sleeping for 8 h. EEG data supported these findings, showing changes in delta power during wakefulness based on perceived sleep duration.

Participants in our study were required to complete a series of at-home exercises that mimicked the main elements of the procedure they experienced during their lab sessions. We know that expectations are fundamental components of placebo effects^54^, and given the pre-study activities common to all three perceived time studies, it seems likely that conditioned expectations^55^ influenced the rates of healing we observed. Future studies that more directly measure and manipulate this aspect of the experimental design are needed to evaluate the contribution of these exercises to the observed effects, which was not possible with this study design. We also did not ask participants to estimate elapsed time within each condition because we found that calling attention to perceived time led pilot participants to infer the timer was manipulated. Thus, we could not evaluate the extent to which rates of healing depended on the degree to which perceived time was successfully manipulated. It could be that the more successful the manipulation, the greater the influence on rates of healing, a possibility that should be addressed in future research.

In terms of other underlying mechanisms specific to our study design, past research indicates moderately warmer temperatures are associated with faster tissue repair^56^. While we did not measure body temperature, and existing research has not consistently observed an effect of subjective durations on body temperatures^57^, it may be that faster subjective time perception results in slightly higher body temperature^58^, thus aiding the healing process. Another mechanism may have been the specific networks of expectations, physiological responses, and beliefs associated with participants’ concepts of time. Some have argued that the behavioral effects of clock time (which is the sense of time we manipulated) depend on the extent to which notions of clock time have been internalized^59^. If internalized concepts of clock time and associated networks of expectations, physiological responses, and beliefs are crucial factors, that would suggest that perceived time should exert far less influence on the physiology of individuals without such internalized notions of clock time, e.g., people living in the villages of Teci and Dalomo on the island of Yasawa in Fiji^60^.

In general, though the list of specific psychological influences on specific health outcomes continues to grow, the factors that shape and amplify those influences remain poorly understood. The field of mind–body health research lacks a broadly agreed upon, overarching theoretical framework to connect known mind–body effects and guide future research, an area ripe for more collaborative research efforts.

## Conclusion

This is the first study to demonstrate that perceived time can affect physical healing, independent of actual time. The psychosocial variables we measured affected healing in a manner consistent with previous research (e.g., the less stressed participants were, the faster they tended to heal; the older participants were, the more slowly they tended to heal), which suggests that healing time expectations may affect actual physical healing times more broadly—a topic we plan to pursue in future in research. These data also suggest that the ways individual minds internalize concepts of time and related expectations and beliefs are not neatly separable from the physiological effects of time. The language of mind–body unity does not undermine the validity of biomedical models that emphasize external factors and lower-level processes. It simply insists on the importance of psychological factors in all aspects of health and wellbeing. Perceptions, expectations, beliefs, and so on are reflected throughout the mind–body and necessarily shape biological and physiological processes. These data and the larger body of research of which they are a part suggest that—at the very least—neglecting the role of the mind when trying to understand any behavior of the body is risky at best and reckless at worst. Language and labels matter, and we count ourselves among the growing chorus of health researchers^61–63^ urging others to start thinking in terms of mind–body “unity.”

### Supplementary Information

Supplementary Figures.

Supplementary Information 2.

Supplementary Information 3.

Supplementary Information 4.

Supplementary Information 5.

Supplementary Information 6.

Supplementary Information 7.

Supplementary Information 8.

Supplementary Information 9.

## Data Availability

All data generated or analyzed during this study are included in this published article and its supplementary information file. Informed consent from all participants and/or their legal guardian(s) for publication of identifying information/images in an online open-access publication and participation was obtained.
