# Author Correction: Physical healing as a function of perceived time

**DOI:** 10.1038/s41598-024-52799-6

**Published:** 2024-01-29

**Authors:** Peter Aungle, Ellen Langer

**Affiliations:** Psychology Department, Harvard University, Cambridge, USA

Correction to: *Scientific Reports* 10.1038/s41598-023-50009-3, published online 17 December 2023

The original version of this Article contained an error in Figure 1, where the scatter points within the violin plots were omitted.

The original Figure 1 and accompanying legend appear below.

**Figure 1:**
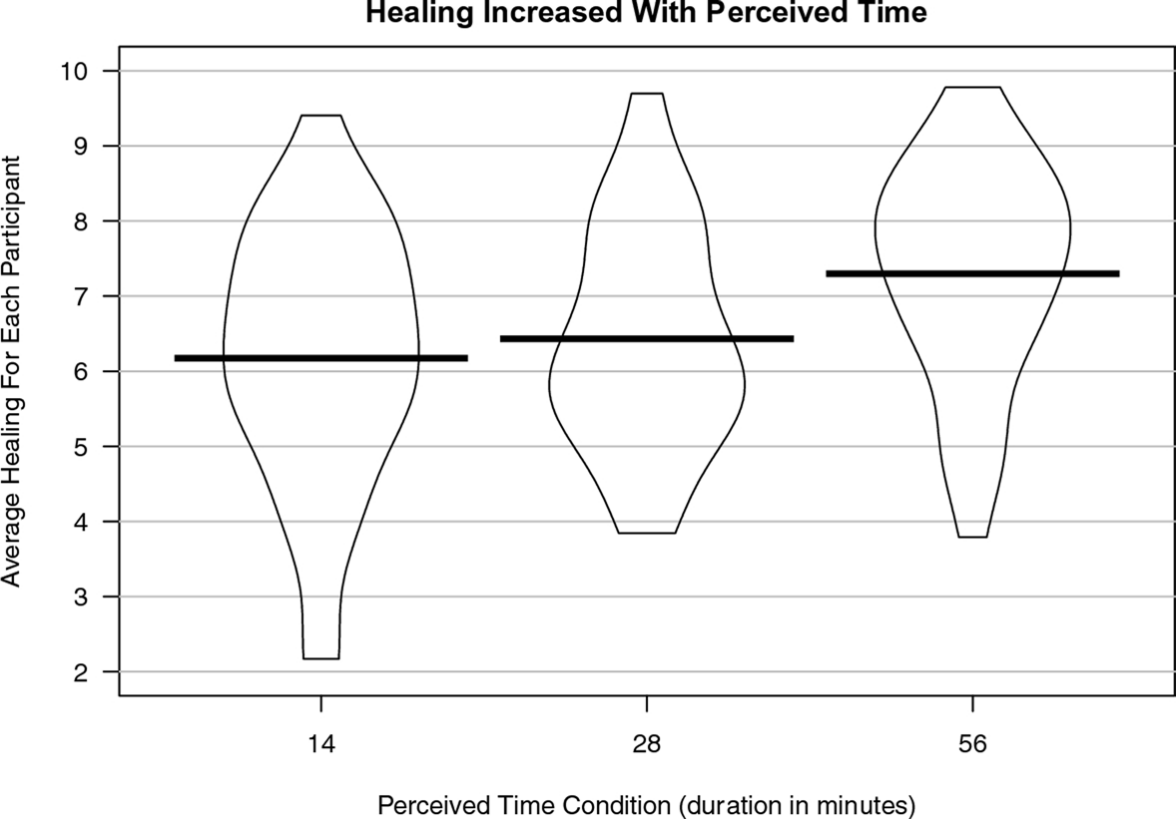
Violin plots of mean healing for each participant within each condition. The more perceived time that passed, the more participants healed on average.

The original Article has been corrected.

