# Supplementary material for: Physical healing as a function of perceived time
Source: Sci Rep. 2023 Dec 17;13:22432. doi: 10.1038/s41598-023-50009-3 (PMC10725481; doi:10.1038/s41598-023-50009-3)

Supplementary Experimental Design Visuals

Supplementary Figure 1: Example cupping kit

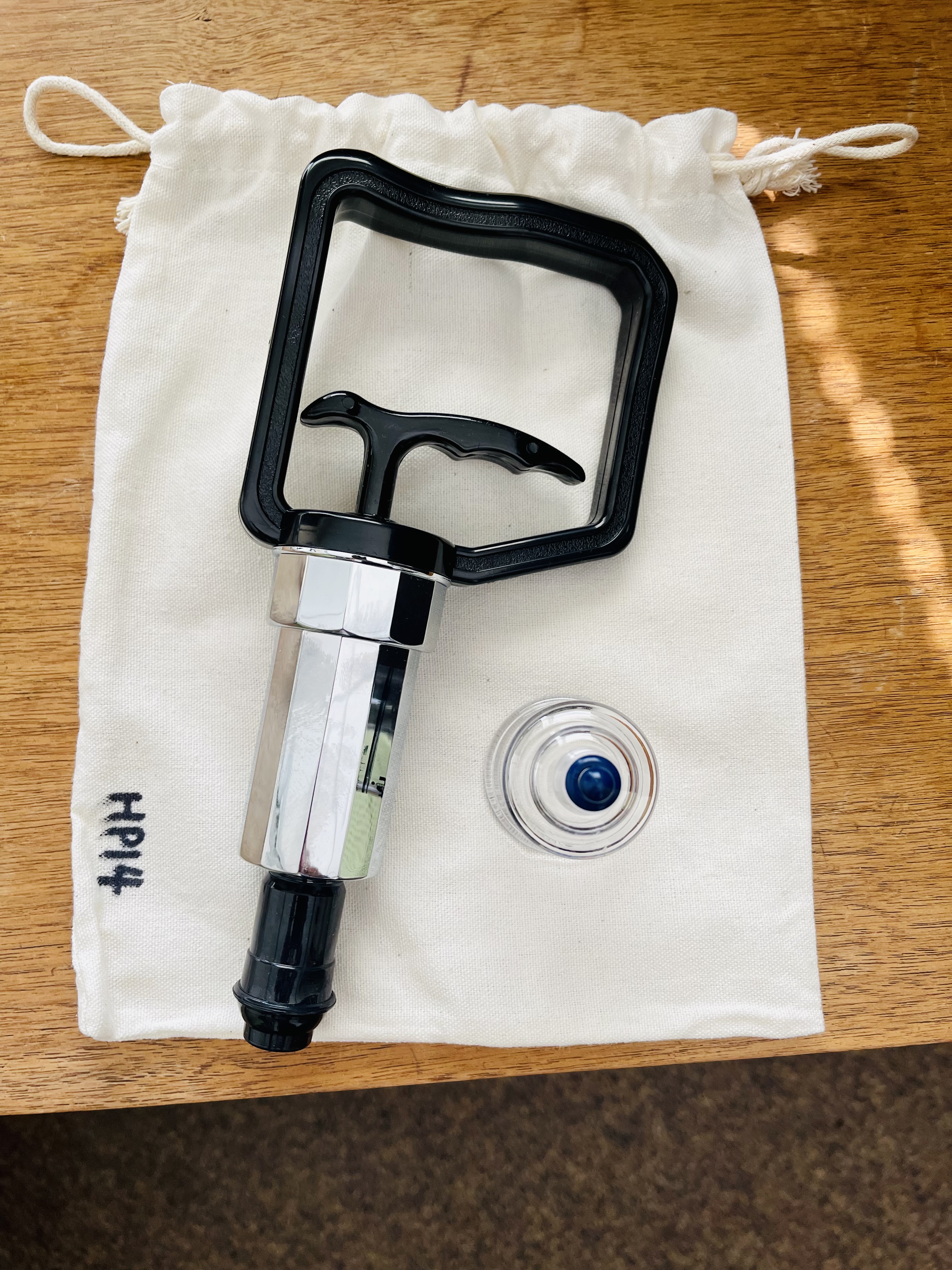


Supplementary Figure 2: Experimenter applying procedure to first author

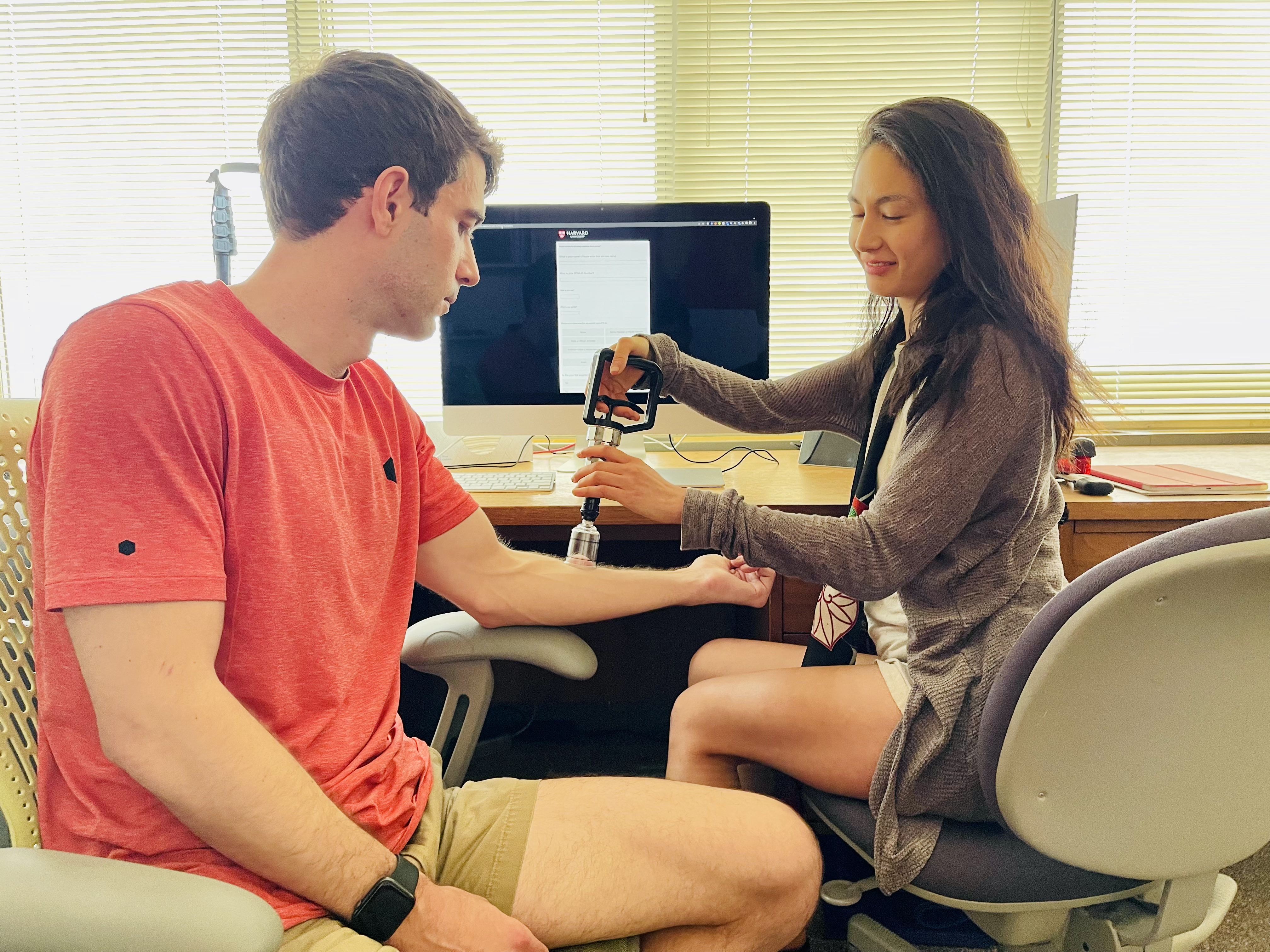


Supplementary Figure 3: Cupping equipment, ring light, light meter (left); rectangular box where participants placed their arms for the picture (blue tape); participant workstation computer (center); tablet timer (right)

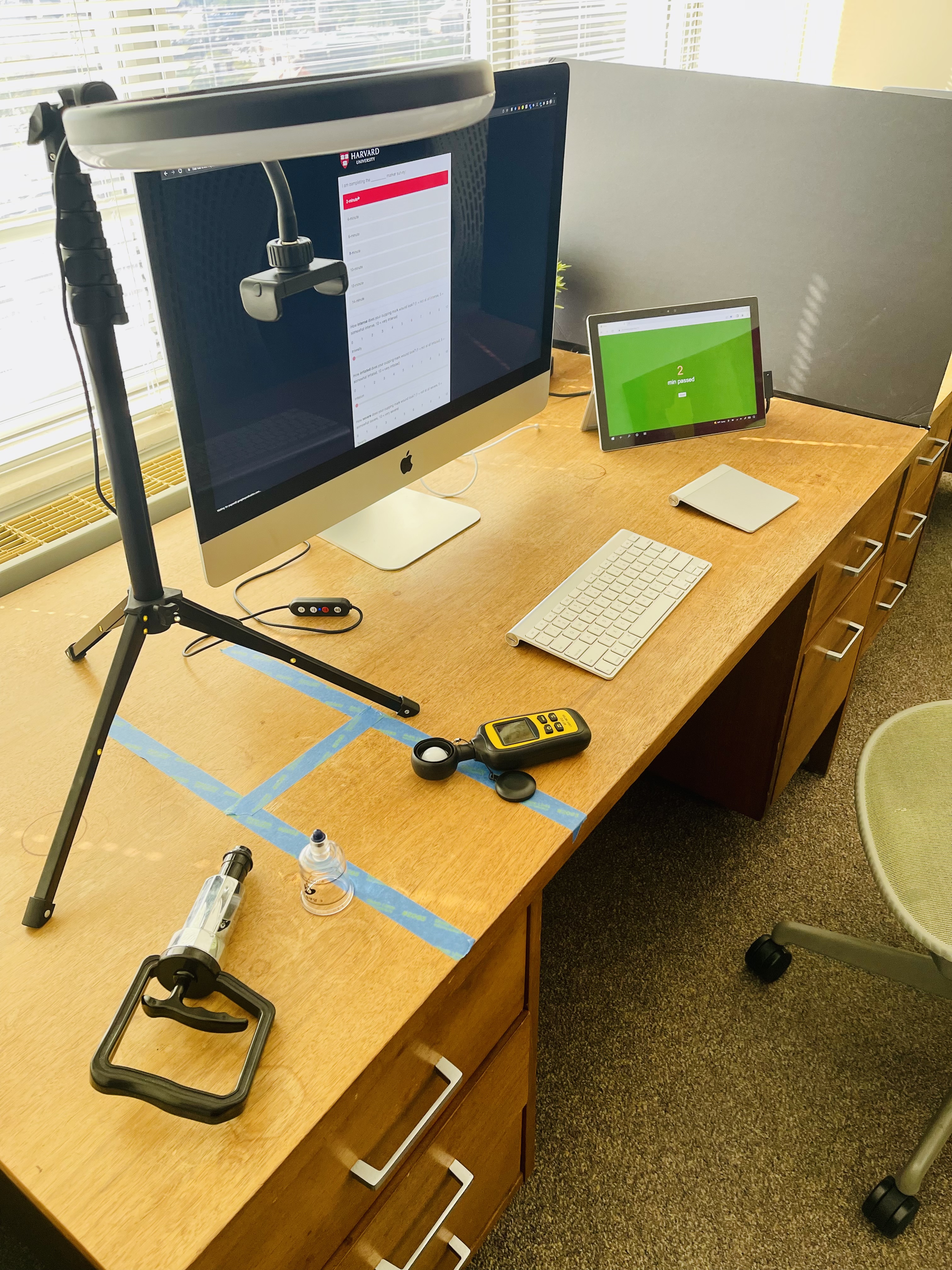


Supplementary Figure 4: Example survey question presented to blind raters on Mturk to assess healing

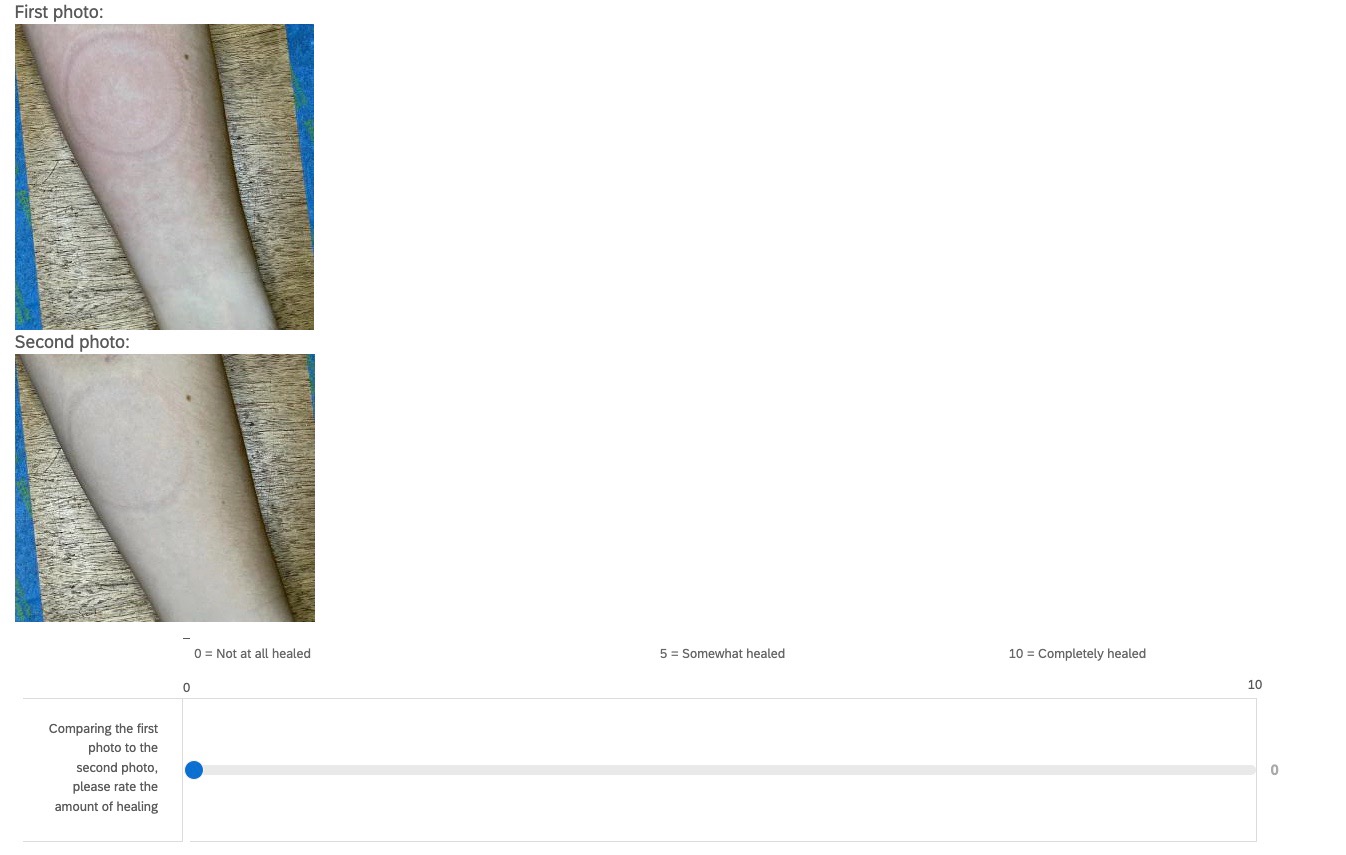

Supplement: Supplementary file 1 — Supplementary Figures. [file 41598_2023_50009_MOESM1_ESM.docx]
